# A robust evaluation of 49 high‐dose‐rate prostate brachytherapy treatment plans including all major uncertainties

**DOI:** 10.1002/acm2.14182

**Published:** 2023-10-14

**Authors:** Andrew Christopher Kennedy, Michael John James Douglass, Alexandre Manuel Caraça Santos

**Affiliations:** ^1^ School of Physical Sciences University of Adelaide Adelaide SA Australia; ^2^ Department of Radiation Oncology Royal Adelaide Hospital Adelaide SA Australia; ^3^ Australian Bragg Centre for Proton Therapy and Research Adelaide SA Australia

**Keywords:** brachytherapy, radiotherapy planning, robust evaluation, uncertainty

## Abstract

**Background:**

Uncertainties in radiotherapy cause deviation from the planned dose distribution and may result in delivering a treatment that fails to meet clinical objectives. The impact of uncertainties is unique to the patient anatomy and the needle locations in HDR prostate brachytherapy. Evaluating this impact during treatment planning is not common practice, relying on margins around the target or organs‐at‐risk to account for uncertainties.

**Purpose:**

A robust evaluation framework for HDR prostate brachytherapy treatment plans was evaluated on 49 patient plans, measuring the range of possible dosimetric outcomes to the patient due to 14 major uncertainties.

**Methods:**

Patient plans were evaluated for their robustness to uncertainties by simulating probable uncertainty scenarios. Five‐thousand probabilistic and 1943 worst‐case scenarios per patient were simulated by changing the position and size of structures and length of dwell times from their nominal values. For each uncertainty scenario, the prostate D_90_ and maximum doses to the urethra, D_0.01cc_, and rectum, D_0.1cc_, were calculated.

**Results:**

The D_90_ was an average 1.16 ± 0.51% (mean ± SD) below nominal values for the probabilistic scenarios; the D_0.01cc_ metric was 2.24 ± 0.90% higher; and D_0.1cc_ was greater by 0.48 ± 0.30%. The D_0.01cc_ and D_90_ metrics were more sensitive to uncertainties than D_0.1cc_, with a median of 79.0% and 84.9% of probabilistic scenarios passing the constraints, compared to 96.5%. The median pass‐rate for scenarios that passed all three metrics simultaneously was 63.4%.

**Conclusions:**

Assessing treatment plan robustness improves plan quality assurance, is achievable in less than 1‐min, and identifies treatment plans with poor robustness, allowing re‐optimization before delivery.

## 1 INTRODUCTION

Radiotherapy aims to deliver sufficient radiation to the target volume to achieve local tumor control while sparing healthy tissue and organs‐at‐risk (OAR) by limiting dose to acceptable thresholds. While the planned dose distribution is optimized to balance these competing objectives, uncertainties in the planning and delivery of the treatment introduce variations in the dose distribution delivered to the patient. The accepted method to account for uncertainties is to place margins around the clinical target volume (CTV) to form a planning target volume (PTV) such that the CTV maintains dose coverage for 90% of patients.^1^ However, margins primarily account for variations caused by geometric uncertainty. In brachytherapy (BT), non‐geometric uncertainties, for instance, source activity and source dwell‐time‐precision, can substantially impact the dosimetric outcome. Since the BT source is a stepping source with a steep‐dose gradient, an inhomogeneous dose distribution results, further reducing margin effectiveness. In some BT techniques, there are minimal or no margins placed around the CTV due to high immobilization, the steep‐dose gradient, and consistent patient positioning for the entire treatment^2^, ^3^, ^4^; consequently, the effects of uncertainties are thought to have minimal impact on outcomes. For these reasons, evaluating the dosimetric impact of major uncertainties on a treatment plan is beneficial to delivering an acceptable treatment.^5^

Robust evaluation quantifies a treatment plan's sensitivity to failure of the clinical objectives due to uncertainty. A lower sensitivity indicates a greater likelihood of delivering a clinically‐acceptable plan, hence is more robust. Techniques to assess the robustness begin with simulating uncertainty scenarios, which involves changing the nominal plan to simulate possible dosimetric outcomes and recalculating the dose.^6^ Robust evaluation is a critical part of intensity‐modulated proton therapy (IMPT) planning^7^, ^8^ and has been applied to intensity‐modulated radiotherapy (IMRT).^6^

Methods for selecting the uncertainty scenarios to simulate are broadly categorized into a worst‐case or probabilistic approach. In the worst‐case approach, plan changes are chosen from possible extreme uncertainty values.^6^, ^7^, ^9^ A plan's robustness is assessed by comparing the worst‐case dosimetric outcomes with alternative plans for the same patient or against historical data. A probabilistic approach involves random sampling of probability distributions for each uncertainty,^6^, ^7^, ^9^ with robustness also assessed by comparisons; however, the probability of dosimetric outcomes is also quantified.

Summarizing the robustness of a treatment plan has been reported using both dose‐volume histograms (DVH) and 3D dose distributions. The spread of DVHs, from uncertainty scenarios, around the nominal plans' is a measure of robustness as it indicates the spread in the dose distribution caused by uncertainty. A tighter spread implies uncertainty causes a lower probable change in the DVH; consequently, the delivered outcome is more predictably closer to what is planned and more robust. Standard deviations, percentiles, or ranges can be used to measure spread at particular doses or volumes representing the objectives.^6^ Alternatively, the DVH metrics can be reported as the minimum, maximum, or mean values from all scenarios. Another robustness measure is the percentage of plans that pass the clinical objectives from all simulated scenarios. In 3D, robustness can also be represented by voxel‐wise minimum, maximum, or mean doses from all scenarios^6^, ^7^ and identifies locations of concern within the patient's anatomy.

Optimizing the robustness of a treatment plan as part of the planning process is a logical progression on from robust evaluation. It seeks to find the most robust plan by incorporating a robustness measure into the optimizer's objective function/s.^6^, ^8^

Within HDR prostate BT, robust optimization and evaluation have been demonstrated using limited uncertainty sources. Balvert et al.^10^ created a min‐max worst‐case optimization method and applied it to three patients planned on ultrasound images and three on CT. Only inter‐ and intra‐observer contouring uncertainty were considered, changing the prostate contour by 2.3–4.4 mm depending on direction. The authors found more robust plans than the TPS optimiser.^10^ Van der Meer et al.^11^ developed a robust optimization algorithm but considered just the uncertainty due to the choice of contour interpolation setting in the TPS.

Poder and Whitaker^12^ evaluated the robustness of plans to catheter displacements of 3, 7, and 14 mm in the inferior direction for 10 patients, finding changes in V_100_ for the prostate target of 0.788 ± 0.751%, −2.803 ± 2.516%, and −18.69 ± 5.197%. In more recent work, Poder et al.^13^ simulated source positioning errors in 16 patient plans from different clinics and compared their robustness. They shifted all catheters simultaneously in 1 mm increments from −6 to 6 mm in the inferior‐superior directions, and, with the same magnitudes, they shifted only the three most heavily weighted catheters simultaneously in the anterior‐posterior and lateral directions. The authors found that inferior‐superior shifts were more considerable for the prostate V_100_ than shifts in the lateral or anterior‐posterior directions. One clinic failed the V_100_ metric by a 1 mm shift, but all others started to fail at a 6 mm magnitude in either direction. Further, they reported that the maximum dose to the urethra had low sensitivity to shifts in inferior‐superior directions but was sensitive to shifts in the lateral and anterior‐posterior directions. Only source positioning uncertainties were considered, and all applied movements to dwells were in the same direction.

In HDR prostate BT, robust optimization and evaluation methods have considered one or a few uncertainties that result in one type of change to the nominal plan, which limits the accuracy of the measured robustness. Further, methods so far have been limited to worst‐case approaches, neglecting the benefit of measuring the likelihood of dosimetric outcomes inherent in a probabilistic approach.

The impact of individual uncertainties on the dose distribution delivered to patients has been well‐researched in the literature, and Kirisitis et al.^14^ have summarized the work and provided examples of achievable uncertainty budgets for some techniques. However, a complete assessment of the impact of uncertainties on individual patient plans before treatment delivery has yet to be demonstrated.

Our previous work^15^ illustrated a framework for robust evaluation in HDR prostate BT on one patient plan. It included the most substantial uncertainties, using probabilistic and worst‐case approaches. The current paper aims to evaluate the framework on a larger patient cohort and form a database of robustly evaluated treatment plans giving the ability to assess and compare the impact of uncertainties on future treatment plans.

## 2 METHOD

### 2.1 Patient selection

Forty‐nine patient plans were retrospectively selected randomly from a cohort of patients receiving HDR prostate BT in a single fraction as a boost to external beam radiotherapy. A live‐ultrasound‐planning technique was used with patients receiving general anesthetic and placed in the lithotomy position for the entire procedure. The average prostate volume was 34 ± 14 cc [11.8, 68.6] (mean ± SD; [min, max]), with an average of 122 ± 36 [50, 190] dwell points, average total dwell time of 500 ± 130 s [255, 810], and 17 [11, 21] median needles. Five patients were prescribed a dose of 15 Gy, and the remainder 16 Gy. The clinical objectives for the patient cohort are available in supplemental material A, Table A1. All treatment plans were created in Vitesse v4.0.1 (Varian Medical Systems, Palo Alto, USA).

### 2.2 Robust evaluation simulations

The robust evaluation method described in previous work^15^ has been developed further to run on a graphics‐processing unit (GPU). In this method, 14 different uncertainties in the HDR prostate BT technique were found to cause five common changes to the structures (prostate, urethra, rectum, dwell points) in the nominal plan and cause variation to dwell times. This allows 14 uncertainties to be simulated in each uncertainty scenario by making six changes to the original plan. These six changes are referred to as parameter variables in this method. The value for each parameter variable in a scenario is chosen based on the robust evaluation technique, probabilistic or worst‐case. Table 1 describes the parameter variables' action on the nominal plan and corresponding magnitudes. Where possible, these magnitudes were obtained from literature.^15^

**TABLE 1:** Parameter variable changes to the nominal treatment plan.

Parameter variable	Treatment plan target	Treatment plan change description	Probabilistic description and values^a^	Worst‐case description and values^b^
1	Dwell points	Dwell points move along the needle in a superior‐inferior direction.	Random movement per needle, all dwell points in a needle move by the same amount. *μ* = 0, *SD* = 1.50 mm	All the dwells in all the needles move in the same direction. [−2.47, 0, 2.47] mm
2	Prostate contour	Prostate contour expands or contracts in a radial direction from the 3D geometric center, with the same magnitude applied to all 3D contour points.	*μ* = 0, *SD* = 1.00 mm	[−1.64, 0, 1.64] mm
3a	Urethra contour	The contour expands or contracts from the 2D geometric center in each slice. The same value is applied to all contour points, and no change occurs in the superior‐inferior direction.	Urethra: *μ* = 0, *SD* = 0.25 mm	Urethra: [−0.41, 0, 0.41] mm
3b	Rectum contour		Rectum: *μ* = 0, *SD* = 0.50 mm	Rectum: [−0.82, 0, 0.82] mm
4a	Dwell points	Dwell points move in transverse slices (2D movement)	Random movement per needle, and all dwell points in a needle move by the same amount. *μ* = 0, *SD* = 1.50 mm	Prostate/Urethra worst‐cases: All dwell points move out or in from the prostate 2D geometric center in each slice. [−2.47, 0, 2.47] mm
4b				Rectum worst‐cases: All dwell points move posteriorly. [−2.47] mm
5a	Dwell times	Dwell times change by a fixed percentage.	Percentage: random per scenario, but the same for all dwells *μ* = 0, *SD* = 4.4%	Percentage: same for all dwells [−7.2, 7.2] %
5b		Dwell times change by a fixed constant amount.	Constant: random per dwell point *μ* = 0, *SD* = 0.060 s	Constant: same for all dwells [−0.099, 0.099] s
6a	Prostate, Urethra contours and Dwell points	Prostate + urethra + dwell points move laterally and anteriorly‐posteriorly.	Lateral: *μ* = 0, *SD* = 0.10 mm	Rectum worst‐cases: Lateral: [−0.16, 0, 0.16] mm
6b			Anterior‐Posterior: *μ* = 0, *SD* = 0.50 mm	Anterior‐Posterior: [−0.82, 0, 0.82] mm

*Note*: The table is a summary of the changes (parameter variables) made to a nominal treatment plan to simulate possible uncertainty scenarios delivered to HDR prostate brachytherapy patients. Included is a description of the change to the nominal treatment plan for two approaches in calculating plan robustness, probabilistic and worst‐case, and the values used for each change. *μ* is the mean of the probability density distribution, *SD* is the corresponding standard deviation.

^a^ Normal distribution, sampled within 90% CI.

^b^ Values from the 90% CI limits of the normal distribution from the probabilistic method.

In the probabilistic case, a unique normal distribution for each parameter variable was randomly sampled within 90% CI. Each normal distribution was formed by adding component uncertainties in quadrature, and 5000 uncertainty scenarios were completed for each nominal plan.

The worst‐case scenarios were conceptualized from expert knowledge of the movements likely to cause extreme DVH metric values,^15^ and where these differed from the probabilistic method, are described in Table 1. The worst‐case values used for each parameter variable were defined to be 90% CI limits from the six probabilistic normal distributions (and zero when appropriate), and all values are shown in Table 1. Values were selected to simulate the worst‐cases for the prostate and urethra (and also assessing the effect on the rectum) by generating all possible combinations of worst‐case values from Table 1 for parameter variables 1, 2, 3a, 3b, 4a, 5a, and 5b. The combinations that had 5a and 5b of [−7.2%, +0.099s] and [+7.2%, −0.099s] were excluded since they would have the opposite effect on dose and so will not lead to the worst‐case scenarios. This led to 486 worst‐case uncertainty scenarios. To obtain the worst‐cases for the rectum (and possibly the other two structures), all combinations of worst‐case parameter variables 1, 2, 3a, 3b, 4b, 5a, 5b, 6a, and 6b were created, with the same exceptions for 5a and 5b as before. The total number of worst‐case uncertainty scenarios grew to 1944 with an additional 1458 being added.

### 2.3 Robustness measures

The dose was calculated using the TG‐43 formulisation^16^, ^17^ for all uncertainty scenarios, and the corresponding DVH curves were obtained. Three DVH metrics were selected to quantify plan robustness, the minimum dose to the hottest 90% of the prostate (D_90_ ≥ 100%), the maximum dose to any 0.01 cc of the urethra (D_0.01cc_ ≤ 115%), and the maximum dose to any 0.1 cc of the rectum (D_0.1cc_ ≤ 13 Gy). A measure of hotspots to the prostate (V_150_ ≤ 40% or V_200_ ≤ 10%) was not considered since they account for hotspots to the urethra due to uncertainties, and the robust evaluation simulates these possibilities directly. The three DVH metrics were selected through consultation with experienced medical physicists involved in the planning procedure. The values for D_0.01cc_ and D_0.1cc_ are expressed as a percentage of the objective to compare patients with 15 and 16 Gy prescriptions.

In the probabilistic method, robustness was quantified by the percentage of uncertainty scenarios that passed the plan objectives, metric pass‐rates; and both the standard deviation, (D*_x_*)_SD_, and mean, (D*_x_*)_mean_, of each DVH metric, *x*. For the worst‐case approach, the worst‐case DVH metric values were used to quantify robustness.

The sensitivity of DVH metrics to change from individual parameter variables was investigated by changing each parameter variable in isolation and calculating the change in each DVH metric.

Two patient plans with low robustness were investigated further by calculating their voxel‐wise minimum, maximum and mean values; one from 1000 probabilistic scenarios and the other from simulated worst‐case scenarios. The dose for every uncertainty scenario was calculated at all points in a 3D dose grid, which enclosed the three contoured organs. All parameter variables were involved in obtaining the voxel‐wise values except changing the contours of the three structures since they do not contribute to a change in voxel‐wise dose.

The algorithm was programmed in Python 3.8.15^18^ and accelerated on an NVIDIA GeForce GTX 1070 Mobile GPU using the CuPy 10.6.0 package^19^ and the Cuda toolkit 10.2.89.^20^

## 3 RESULTS

### 3.1 Probabilistic robust evaluation

**FIGURE 1:**
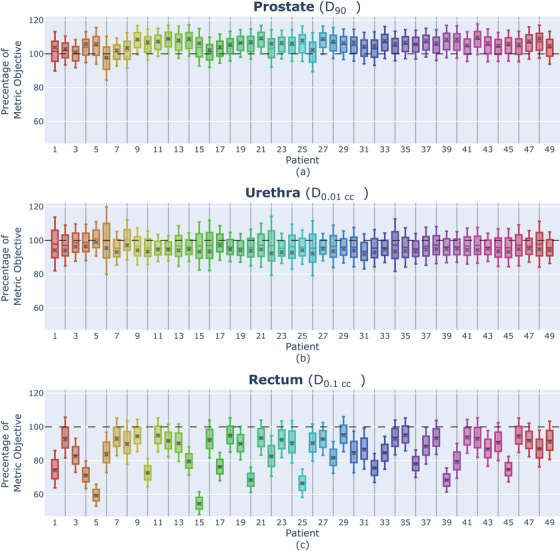
**Nominal treatment plan dose metric distributions from 5000 uncertainty scenarios**. Box plots from 5000 probabilistic uncertainty scenarios for 49 patients' treatment plans, with the grey cross indicating the nominal metric value, the mean of the distribution, (D*_x_*)_mean_, being the center line of the rectangular boxes, the top and bottom of the rectangular boxes showing standard deviation, (D*_x_*)_SD_, and the upper and lower fences representing the 95% confidence interval, respectively. (a) D_90_ of the prostate target; (b) the maximum dose to the urethra, D_0.01cc_; (c) the maximum dose to the rectum, D_0.1cc_. The black dashed lines indicate the dose metric objectives. All values on the vertical axes are given as the percentage of the dose metric objective value.

Across all nominal plans, (D_0.01cc_)_mean_ was, on average, 2.24 ± 0.90% (mean ± SD) higher than the nominal values, and the value for two patient plans was above 115%, see Figure 1. For (D_90_)_mean_, one patient plan was below the constraint, and the average decrease was 1.16 ± 0.51%. All treatment plans had (D_0.1cc_)_mean_ below the 13 Gy constraint; the average difference was an increase of 0.47 ± 0.30%. The maximum dose to the urethra had the largest variation, with the average (D_0.01cc_)_SD_ across all nominal plans of 5.7 ± 1.3% [3.8, 10.2] ([min, max]), indicating it was the least robust to uncertainties. The prostate dose metric had the lowest average variation with (D_90_)_SD_ of 4.76 ± 0.51% [4.17, 6.60], and the average (D_0.1cc_)_SD_ of the rectum was 5.12 ± 0.78% [3.35, 6.51].

**FIGURE 2:**
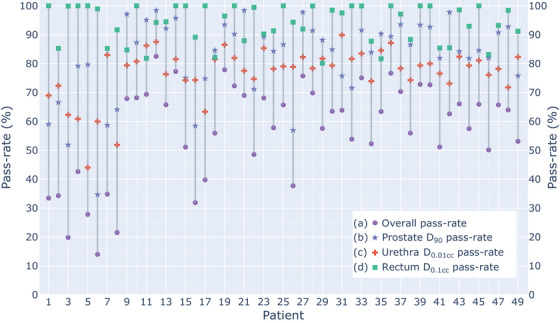
**Dose metric pass‐rates**. Percentage of probabilistic uncertainty scenarios from 5000 that passed the dose metrics for (a) all three DVH metrics simultaneously, (b) the D_90_ > 100% of the prescribed dose for the prostate, (c) the maximum dose to the urethra, D_0.01cc_ < 115% of the prescribed dose, and (d) the maximum dose to the rectum, D_0.1cc_ < 13 Gy.

Pass‐rates for each of the three DVH metrics of interest are shown in Figure 2. The maximum dose to the urethra had the lowest pass‐rate among the three DVH metrics with a median pass‐rate of 79.0% [74.3, 82.0] ([1st quartile, 3rd quartile]), also indicating it was the least robust to uncertainties. The prostate D_90_ had a median pass‐rate of 84.9% [75.8, 92.2], and the D_0.1cc_ of the rectum had the highest robustness out of the three metrics, with a median pass‐rate of 96.5% [88.4%, 100.0%]. Overall plan robustness, simultaneously passing all three DVH metrics in a single uncertainty scenario, is indicated by the purple circles in Figure 2, and the median from all 49 nominal plans was 63.4% [50.1%, 69.0%]. Patient 6 had the lowest overall pass‐rate of 14.0%, the lowest D_90_ pass‐rate, and the third lowest urethra D_0.01cc_ pass‐rate. This is explained by the small size of the prostate (13.3 cc) and three heavily weighted dwell positions (18.3, 21.7, 20.8 s) close to the surface of the prostate that causes the D_90_ metric to be sensitive to failure when moved relative to the small dimensions of the prostate.

### 3.2 Worst‐case robust evaluation

**FIGURE 3:**
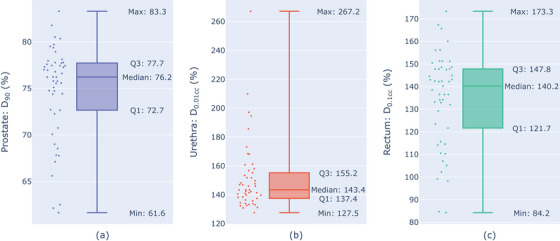
**Worst‐case dose metric values**. The most extreme DVH metric values from 49 nominal treatment plans when worst‐case uncertainty values were simulated for (a) D_90_ of the prostate target, (b) the maximum dose to the urethra, D_0.01 cc_, and (c) the maximum dose to the rectum, D_0.1cc_. Values are stated as the percentage of the dose metric objective.

The DVH metrics resulting from the worst‐case uncertainty scenarios are shown in Figure 3. The parameter variable changes which caused the lowest D_90_ values for 43 patient plans occurred when the dwell times decreased by 7.2% and then 0.099 s (except patient 6); prostate contour expanded by 1.64 mm; dwell points moved out 2.47 mm from the prostate geometric mean line; and, dwell points moved superiorly or inferiorly along needles by 2.47 mm. The remaining patient plans (16, 26, 34, 38, 46, 47) had the worst D_90_ values with the same parameter variable changes, except all dwell points moved posteriorly 2.47 mm instead of radially outward. For the urethra D_0.01cc_, all but one highest value occurred with dwell times increasing by 7.2% and then by 0.099 s (except patient 6); urethral contour contracting by 0.411 mm; and dwell points moving radially inward by 2.47 mm. The highest values for the rectum D_0.1cc_ were from dwell times increasing by 7.2% and then by 0.099 s; the rectum contour expanding by 0.822 mm; the prostate, urethra, and dwell points moving posteriorly by 0.82 mm; and, all dwell points moving an additional 2.47 mm posteriorly.

### 3.3 Parameter variable sensitivity

The sensitivity of DVH metrics to each parameter variable's worst‐case change is shown in Table 2. The dwell time percentage, parameter variable 5a, affected the DVH metrics consistently, with a maximum change across all nominal plans of 7.27% for D_90_, 7.36% for D_0.01cc_, and 7.34% for D_0.1cc_. Dwell points moving towards the prostate center line and posteriorly, 4a and 4b in Table 2, resulted in large changes to the three metrics, with a maximum decrease of 29.4% for D_90_ and maximum increases of 140% for D_0.01cc_ and 28.5% for D_0.1cc_.

**TABLE 2:** Dose‐volume metric sensitivities to parameter variable changes.

Parameter variable	Change value	Prostate: D_90_ (%)	Prostate: V_150_ (%)	Urethra: D_0.01 cc_ (%)	Rectum: D_0.1 cc_ (%)
1: All dwell points move along needles in the same direction	−2.47 mm	−0.35 ± 0.89	−1.1 ± 1.8	0.06 ± 0.60	−1.58 ± 0.95
	2.47 mm	−2.7 ± 2.8	−3.3 ± 2.0	0.78 ± 0.88	1.6 ± 1.0
2: Prostate expansion or contraction from the 3D geometric mean point (Same value applied to all 3D contour points)	−1.64 mm	1.9 ± 1.8	−2.6 ± 6.3	0	0
	1.64 mm	−4.5 ± 2.2	−5.9 ± 4.2	0	0
3a: Urethra expansion or contraction from the geometric mean in each slice (Same value applied to all contour points per slice)	−0.411 mm	0	0	−0.80 ± 0.42	0
	0.411 mm	0	0	0.96 ± 0.50	0
3b: Rectum expansion or contraction from the geometric mean in each slice (Same value applied to all contour points per slice)	−0.822 mm	0	0	0	−5.36 ± 0.79
	0.822 mm	0	0	0	5.92 ± 0.96
4a: Dwell points move out (+) or in (‐) from the prostate 2D geometric center from each slice	−2.47 mm	11.4 ± 4.7	79 ± 18	33 ± 18	4.7 ± 3.3
	2.47 mm	−17.7 ± 3.0	−54.7 ± 7.4	−18.4 ± 2.6	−4.2 ± 3.3
4b: Dwell points move posteriorly	2.47 mm	−3.9 ± 4.1	−6.0 ± 5.9	3.8 ± 4.5	19.7 ± 3.7
5a: Dwell times change by a fixed percentage.	−7.2%	−7.2353 ± 0.0085	−22.4 ± 2.5	−7.237 ± 0.042	−7.234 ± 0.033
	7.2%	7.230 ± 0.019	26.5 ± 3.7	7.219 ± 0.054	7.243 ± 0.039
5b: Dwell times change by a fixed constant amount.	−0.099 s	−2.79 ± 0.46	−8.6 ± 1.9	−2.65 ± 0.41	−2.76 ± 0.57
	0.099 s	2.77 ± 0.44	9.5 ± 2.2	2.7 ± 0.42	2.78 ± 0.58
6: Prostate + urethra move posterior	−0.822 mm	0	0	0	−5.30 ± 0.78
	0.822 mm	0	0	0	5.81 ± 0.96

*Note*: The percentage change of dose‐volume metrics from their nominal values due to single parameter variable worst‐case changes. Percentage changes from 49 patient plans are given as the mean ± SD. Highlighted are the three highest contributors to the respective dose‐volume metric not meeting the clinical objective.

The probability of parameter variable four occurring in the worst‐case is very low since all needles are required to move radially outwards, or all posteriorly, by 2.47 mm. Each needle has a low probability of this magnitude change within this model. Due to this and the DVH metrics having a large sensitivity to parameter variable four, the sensitivity of the metrics was also investigated using 1000 probabilistic uncertainty scenarios. The prostate D_90_ decreased by an average of 0.8 ± 2.3% (mean ± SD) with a maximum of 12.7%. The urethra D_0.01cc_ increased by 2.0 ± 4.7% (max: 100% for patient 6), and the rectum D_0.1cc_ increased by 0.4 ± 2.9% (max: 17.0%).

### 3.4 Voxel‐wise dose distributions

**FIGURE 4:**
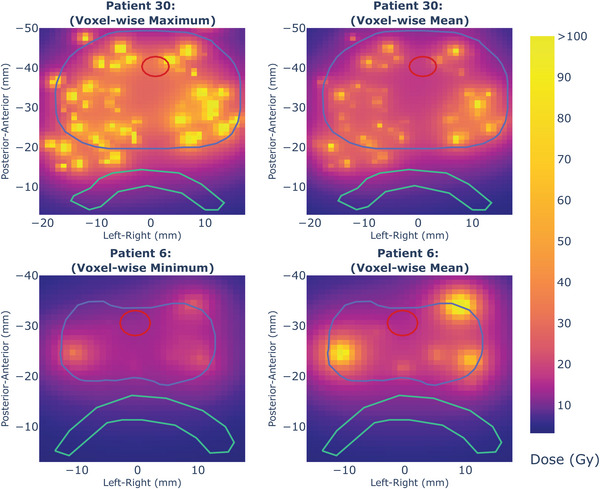
**Voxel‐wise dose distributions**. The voxel‐wise maximum and mean dose distributions resulting from the worst‐case uncertainty simulations for patient 30′s treatment plan and the voxel‐wise minimum and mean dose distributions for patient 6 resulting from 1000 probabilistic uncertainty scenarios. The prostate is shown as a blue contour, the urethra is indicated in red, and the contoured part of the rectum is green. A central prostate slice is shown for patient 30 with a high dose indicated by the orange voxels to the top right of the urethra in the images. For patient 6, a central prostate slice is also shown, and low‐dose voxels in the top‐left of the prostate in the images are indicated in purple. Voxel‐values have been limited to 100 Gy.

For patient 30, the worst‐case scenario for the urethra resulted in the value of D_0.01cc_ being 267.2%, shown in Figure 3(b). Investigating this further using the voxel‐wise mean and maximum dose distributions, shown in Figure 4, indicates a mean dose of 27.2 Gy and a maximum dose of 86.4 Gy to the same 1 mm‐cubed voxel within the urethral contour from a dwell point moving close to the urethra, which is the likely cause of the extreme DVH metric value. Similar levels of dose were also detected in adjacent voxels. Patient 6′s treatment plan had the lowest pass‐rate for D_90_, as seen in Figure 2. The voxel‐wise mean dose for this nominal plan, Figure 4, shows low‐dose regions receiving 10.9 Gy and voxel‐wise minimum doses of 8.8 Gy. This information would guide further adjustment of the nominal treatment plan to increase robustness.

### 3.5 Algorithm runtime

The mean calculation time for a single uncertainty scenario was 0.32 ± 0.12 s. The largest average runtime was 0.57 ± 0.10 s for patient 35, with 20 needles and a prostate volume of 68.6 cc. The lowest average runtime was 0.158 ± 0.037 s for patient 6, with 11 needles in the plan and a prostate volume of 13.3 cc. The largest component of the runtime involved the TG‐43 dose calculation.  Larger prostate volumes and total dwell points in a treatment plan result in more element‐wise array calculations, increasing processing time and memory resources needed. The algorithm runtime increases approximately linearly with prostate volume and active dwell points.

The number of required uncertainty scenarios further impacts the total time to perform a robust evaluation. For the worst‐case procedure, 15 uncertainty scenarios resulted in the worst metric values across all nominal plans. In the probabilistic technique, the level of statistical uncertainty in robustness is dictated by the total number of uncertainty scenarios used (see supplemental material B, Figure B1). A statistical uncertainty lower than 5% in the robustness measures is first obtained for the overall pass‐rate using 105 uncertainty scenarios, which gives a median statistical uncertainty of 4.6% and a percentile 95% CI of [3.7%, 5.0%]. The median statistical uncertainty for the standard deviation of the DVH metrics were: Δ(D_90_)_SD_ = 0.29% [0.26%, 0.39%], Δ(D_0.01cc_)_SD_ = 0.54% [0.26%, 1.26%], and Δ(D_0.1cc_)_SD_ = 0.36% [0.23%, 0.47%].

## 4 DISCUSSION

Both probabilistic and worst‐case techniques communicate essential aspects of a treatment plan's robustness. The probabilistic algorithm presents information about the probability of clinical objectives being met, whereas the worst‐case measures the extreme limits of what could be delivered to a patient, however unlikely. Each measure of robustness becomes meaningful when evaluated in the context of a historical set of robustly evaluated treatment plans, which provides consistency with the robustness measurement used. In the authors' opinion, the most valuable measurement of plan robustness is the overall pass‐rate from the probabilistic algorithm since the number of possible delivered scenarios ending in the patient receiving a clinically acceptable plan is of most interest. To use the historical set of robustness data, an acceptable threshold should be derived at the clinic level, which, when reached, would trigger further investigation of the cause and inform plan re‐optimization. Ideally, the threshold would be based on historical patient outcomes. The pass‐rates and DVH metric robustness statistics, (D*_x_*)_SD_ and (D*_x_*)_mean_, for each structure (prostate, urethra, rectum) inform which structure to target for improvement in robustness. Further, the voxel‐wise minimum, maximum, and mean doses viewed in 2D or 3D (see supplemental material C, Figure C1) highlight specific dwell positions to adjust for improvement in robustness.

Uncertainty magnitudes were obtained from literature as described in previous work^15^ and are technique‐specific but not clinic specific. Applying uncertainties for a specific clinic is critical for the accuracy in simulating uncertainty scenarios and, therefore, the resulting robustness measurement. The algorithm allows flexibility in changing these 14 uncertainty magnitudes, hence the six parameter variables. From Table 2, the two largest contributions to robustness were uncertainties affecting the 2D movement of dwell points (needle reconstruction and intrafraction needle movement) and uncertainties affecting dwell times (TG43 dose calculation, source activity, source dwell time precision). Quantifying these uncertainties within the clinic and employing steps to improve needle reconstruction and source activity measurements would have the most impact on increasing plan robustness.

Furthermore, knowledge of clinic‐specific uncertainties can aid treatment planning. For example, changing the 2D location of the dwell points inwards to the prostate center line, parameter variable 4a in Table 2, by 2.47 mm for patient 30 resulted in the urethral maximum dose metric exceeding the constraint by 167.2%. This highlights the advantage of clinic‐specific knowledge for inter‐ and intra‐observer contouring uncertainties and should be used to limit the weighting of dwell points within this uncertainty distance from the urethra.

A TG‐43 dose calculation was developed as part of the robust evaluation algorithm, and the resulting DVH curves for each structure were compared with the DVH curves from the TPS for all nominal plans. There was an absolute mean difference of 0.105 ± 0.041% (mean ± SD) between the relative volume percentage arrays for the prostate, 0.172 ± 0.092% for the urethra, and 0.062 ± 0.026% for the rectum, confirming the accuracy of the inbuilt dose calculation.

The algorithm's runtime has improved from the previous work^15^ through the implementation of the algorithm on GPU. The algorithm runtime can be split into the time required to make plan changes and the dose calculation. For CPU implementation of the algorithm, the runtime was 0.51 ± 0.16 s (mean ± SD) for making the plan changes and 3.2 ± 1.5 s for calculating the dose from 100 uncertainty scenarios. For the GPU implementation, the plan changes took 0.153 ± 0.040 s and calculating the dose took 0.193 ± 0.088 s. Implemented on CPU, the dose calculation performed at a rate of 16 ± 4.9 thousand dose calculation points per second (mean ± SD), compared to 310 ± 220 thousand dose calculation points per second on the GPU, a 19 times increase in dose calculation points per second. Since 105 probabilistic scenarios are necessary for a statistical uncertainty of better than 5% and 15 worst‐case scenarios are needed to obtain the most extreme DVH metric values, the total runtime is approximately 42 s, a clinically suitable time frame.

The worst‐case magnitudes simulated for dwell positions were less than Podder et al.,^13^ which simulated movements up to 6 mm. From Table 2, lateral and anterior‐posterior movements of dwells impacted the D_90_ metric more than in the inferior‐superior direction; however, Podder et al.^13^ found the opposite for the V_100_ metric of the prostate. This may be explained by the source positioning error Podder et al.^13^ were simulating and only moving the three most heavily weighted catheters in transverse slices, but all catheters moving in the superior‐inferior direction. They noted that all but one department began failing the V_100_ metric at shifts greater than 6 mm in the inferior‐superior direction, well below the 2.47 mm shifts in this study. This might indicate that inferior‐superior shifts become larger than transverse position uncertainty at shifts above 6 mm, but further research is required. Podder et al.^13^ findings were consistent for the maximum dose to the urethra shown in Table 2.

Results obtained for the effect of individual uncertainties on the DVH metrics for the 49 patients' plans are consistent with reported results considering technique, patient numbers in the study, and uncertainty magnitude differences. The prostate boundary expanding by 1.64 mm for a worst‐case scenario decreased D_90_ by 4.5 ± 2.2%, comparable to the 7 ± 11% decrease due to contouring uncertainty reported by Rylander et al.^21^ when comparing US and MR contours of the CTV across 11 patients. They found that contouring and needle reconstruction uncertainties had the average effect of reducing the D_90_ metric. This is equivalent to the findings in Table 2, showing that changes due to parameter variables 1, 2, and 4, on average, caused a reduction in D_90_. Even et al.^22^ reported an average increase of 9.4% (max: 15.5%) for D_10_ of the urethra for eight patients because of needle reconstruction uncertainty in US planning; this is higher than the D_0.01cc_ change of 2.0 ± 4.7% obtained for the urethra across 1000 probabilistic scenarios in the paper. Also, Even et al.^22^ reported a higher average variation in D_1cc_ for the rectum of +4.3%, and in this work 0.4 ± 2.9% was obtained for D0.1 cc. This difference may be due to the use of a spacer (dureseal) applied between the rectum and prostate in the clinic from which the 49 patient plans were obtained.

Future research will involve the development of a robust optimization algorithm expanding on the robust evaluation method presented here. The robust evaluation runtime impacts the number of plans that can be robustly evaluated, and further investigation of methods to reduce this runtime will be critical for a robust optimization to be completed in a clinically suitable time frame. Also, the algorithm could likely be adapted for other treatment sites within BT by adjustments of the coded movements and programming new parameter variable changes which align with relevant uncertainties within that treatment site and technique. The algorithm would also support further research investigating the effects of uncertainty within other HDR prostate BT techniques.

## 5 CONCLUSION

A robust evaluation method was demonstrated on 49 HDR prostate BT patient plans, which was able to perform the evaluation in a clinically acceptable time, under 1 min, and included contributions to plan variations from 14 uncertainties. On average, uncertainty in the technique results in the most likely dosimetric outcome delivered to the patient to be worse than the nominal plan's DVH metrics for the D_90_ of the prostate, D_0.01cc_ for the urethra, and D_0.1cc_ for the rectum, indicating that patients are more likely to receive lower doses to the prostate target and higher doses to the OAR than planned. The largest contributor to a nominal plan's robustness was uncertainty in the 2D transverse location of dwell points and dwell times. Nominal plan robustness to uncertainty indicated by the overall plan pass‐rates ranged from 14.0% to 82.5%, with a median of 63.4%. The distribution of robustness values from the 49 patient plans provides context for judging the robustness values of future nominal plans. Further, the robustness measures presented aid re‐optimization of the plan by highlighting structures of concern and locating dwell points to target to improve treatment plan robustness.

## AUTHOR CONTRIBUTIONS

Andrew Christopher Kennedy: designed the model and method of analysis, collected data, performed analysis, wrote the paper, and revised the paper. Alexandre Manuel Caraça Santos and Michael John James Douglass: substantially contributed to the conception of the work; and its direction in terms of development, analysis, and interpretation. Drafted the paper critically for important intellectual content and final approval of the version to be published. All authors agree to be accountable for all aspects of the work in ensuring that questions related to the accuracy or integrity of any part of the work are appropriately investigated and resolved.

## ETHICS STATEMENT

This study was performed in line with the principles of the Declaration of Helsinki. Approval was granted by the Central Adelaide Local Health Network Human Research Ethics Committee on January 13, 2022, Reference Number 15744.

## CONFLICT OF INTEREST STATEMENT

The authors have no relevant conflicts of interest to disclose.

## Supporting information

Supporting Information

Supporting Information

Supporting Information

## Data Availability

Research data are not available at this time
