# Supplementary material for: A robust evaluation of 49 high‐dose‐rate prostate brachytherapy treatment plans including all major uncertainties
Source: J Appl Clin Med Phys. 2023 Oct 14;25(2):e14182. doi: 10.1002/acm2.14182 (PMC10860441; doi:10.1002/acm2.14182)
Supplement: Supplementary file 1 — Supporting Information [file ACM2-25-e14182-s001.pdf]

Table A1. DVH metrics with constraints that quantify the clinical objectives for the HDR prostate brachytherapy patients considered in this work.

Prostate	Urethra	Rectum
$D_{90} \geq 100\%$<br>$V_{100} \geq 90\%$<br>$V_{150} \leq 40\%$<br>$V_{200} \leq 10\%$	$D_{10} \leq 115\%$<br>$D_{0.01\ cc} \leq 115\%$	$V_{75} \leq 1.0$ cc<br>$D_{0.1\ cc} \leq 13$ Gy
