# Supplementary material for: A robust evaluation of 49 high‐dose‐rate prostate brachytherapy treatment plans including all major uncertainties
Source: J Appl Clin Med Phys. 2023 Oct 14;25(2):e14182. doi: 10.1002/acm2.14182 (PMC10860441; doi:10.1002/acm2.14182)
Supplement: Supplementary file 2 — Supporting Information [file ACM2-25-e14182-s003.pdf]

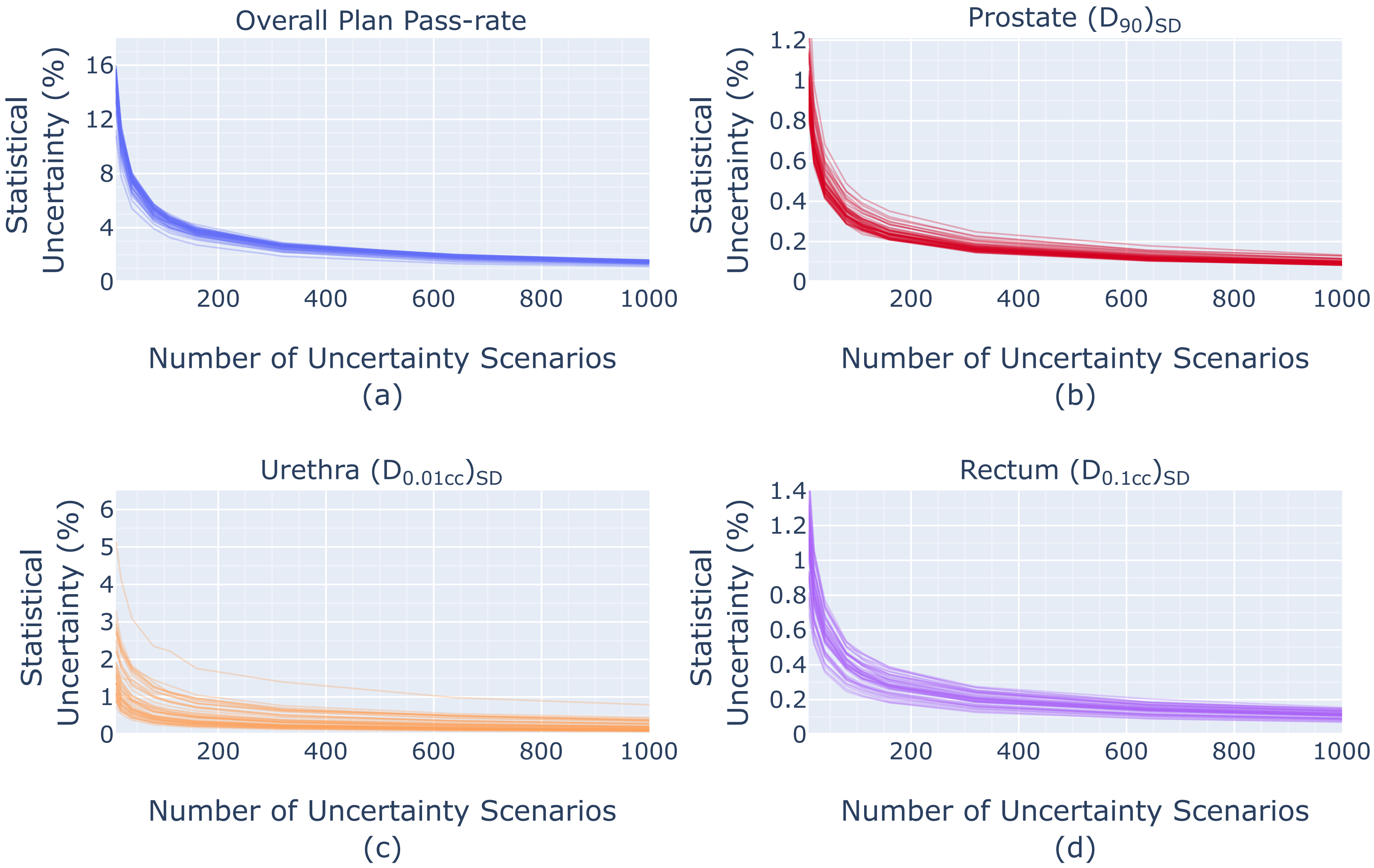

Fig B1: The statistical uncertainty resulting from the choice of the number of probabilistic uncertainty scenarios used to robustly evaluate a nominal treatment plan. Treatment plan robustness was calculated by four measures: **(a)** the overall plan pass-rate percentage, and the standard deviations of **(b)** the prostate D_{90}, **(c)** Urethra $D_{0.01cc}$, and **(d)** rectum $D_{0.1cc}$, the latter three as a percentage of the objectives for the DVH metric. The statistical uncertainty in each of the four robustness measures was quantified by the standard deviation from 1000 robustness samples, each sample of size equal to the *number of uncertainty scenarios* (changed from 10 to 1000) from a population of 5000 uncertainty scenarios, with replacement. The curve is plotted for each of the 49 patient's treatment plans.
