# Supplementary figures and images for: A robust evaluation of 49 high‐dose‐rate prostate brachytherapy treatment plans including all major uncertainties
Source: J Appl Clin Med Phys. 2023 Oct 14;25(2):e14182. doi: 10.1002/acm2.14182 (PMC10860441; doi:10.1002/acm2.14182)

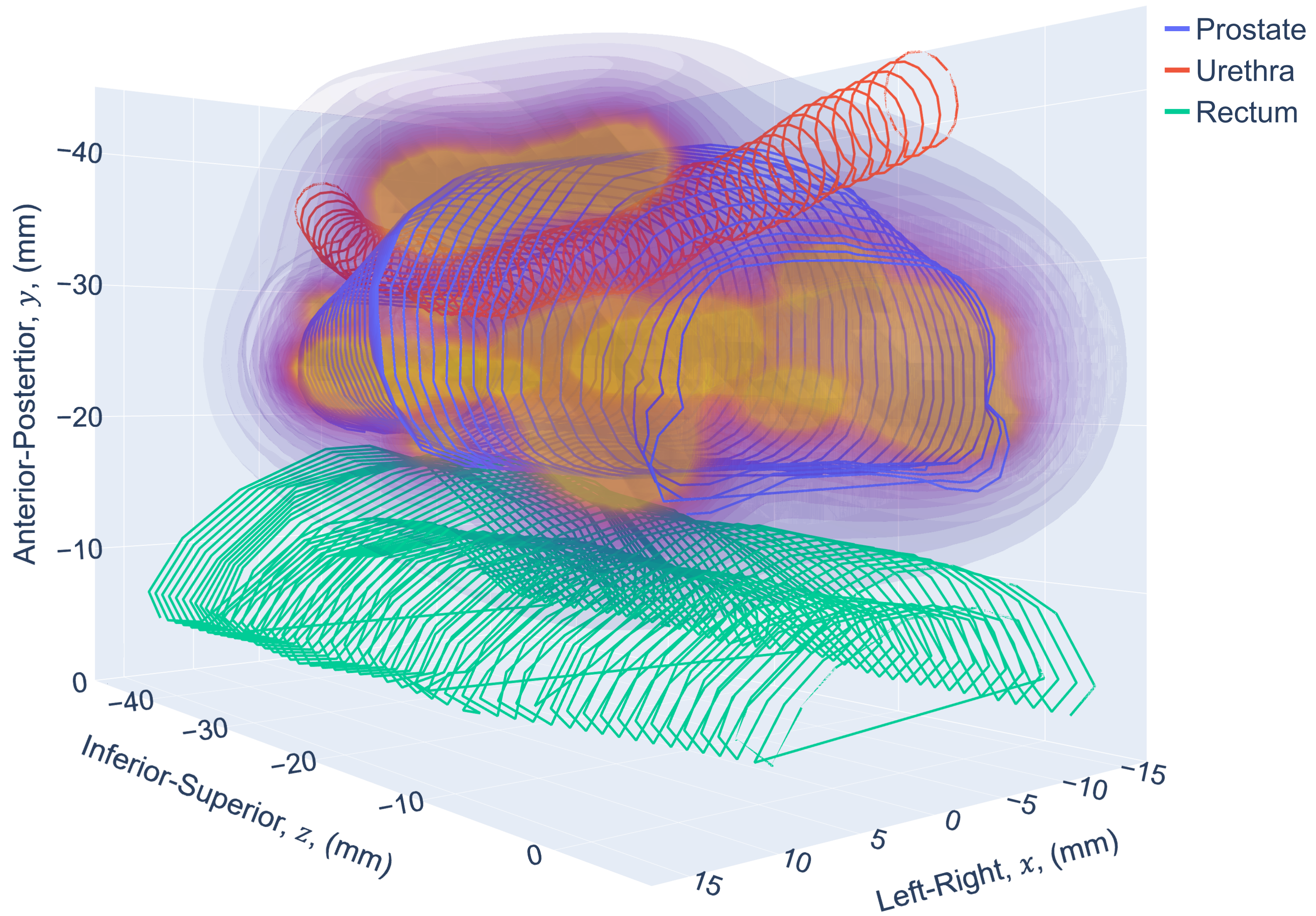

**Fig C1:** The voxel-wise mean dose from 1000 probabilistic uncertainty scenarios for patient 6.

Supplement: Supplementary file 3 — Supporting Information [file ACM2-25-e14182-s002.pdf]
